# New Proposal in the Approach to Brachial Remodeling: The Melfa-Anastasia Arm Laxity System (MAALS)

**DOI:** 10.1093/asjof/ojag150

**Published:** 2026-08-04

**Authors:** Melfa Fabrizio, Anastasia Dafne, Ciranna Gabriele, Melfa Sonia, Nicoletti Giovanni

**Affiliations:** Surgery and Aesthetic Medicine, Mediaging Clinic Center, Palermo and Milan, Italy; Interdepartmental Research Centre for Technologies Applied to Regenerative Medicine and Inductive Surgery (T.A.Me.Ri.C.I.), University of Pavia, Pavia, Italy; Surgery and Aesthetic Medicine, Mediaging Clinic Center, Palermo and Milan, Italy; Interdepartmental Research Centre for Technologies Applied to Regenerative Medicine and Inductive Surgery (T.A.Me.Ri.C.I.), University of Pavia, Pavia, Italy; Surgery and Aesthetic Medicine, Mediaging Clinic Center, Palermo and Milan, Italy; Surgery and Aesthetic Medicine, Mediaging Clinic Center, Palermo and Milan, Italy; Interdepartmental Research Centre for Technologies Applied to Regenerative Medicine and Inductive Surgery (T.A.Me.Ri.C.I.), University of Pavia, Pavia, Italy; Interdepartmental Research Centre for Technologies Applied to Regenerative Medicine and Inductive Surgery (T.A.Me.Ri.C.I.), University of Pavia, Pavia, Italy

## Abstract

**Background:**

Current diagnostic tools for upper arm laxity assessment—including the pinch test and two-dimensional photographic scales—fail to characterize the underlying tissue composition, leading to inappropriate treatment selection and preventable complications.

**Objectives:**

To introduce the Melfa-Anastasia Arm Laxity System (MAALS), a precision medicine framework integrating high-frequency ultrasound (HFUS) biometry with a cylindrical geometric model for objective, compositional assessment of brachial architecture.

**Methods:**

MAALS employs a noncontact stand-off technique (HFUS, 18-22 MHz) to measure superficial adipose tissue thickness (SAT) and triceps muscle thickness (M) at the mid-brachial sentinel point. Two dimensionless indices are derived: the adipose saturation percentage (%SAT = SAT/Rtot × 100) and the muscle filling ratio (MFR = M/Rtot), where Rtot is the total brachial radius calculated from arm circumference.

**Results:**

Based on preliminary anatomically rational thresholds, MAALS defines five phenotypes (0-4): normotrophic (0), adipose hypertrophy (1), mixed morphology (2), pure cutaneous ptosis with critical vascular risk zone (%SAT < 20%, SAT < 4-5 mm; liposuction contraindicated) (3), and sarcopenic/atrophic (4). Each phenotype carries specific therapeutic indications ranging from prevention to regenerative strategies.

**Conclusions:**

MAALS is presented as a conceptual precision medicine framework requiring prospective validation, not as an established clinical standard. By providing objective compositional characterization of brachial architecture, it offers a structured basis for safer, more individualized treatment planning, future multicenter validation studies, and integration with AI-based segmentation technologies.

**Level of Evidence: 5 (Therapeutic):**

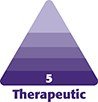

Upper arm rejuvenation has seen advancements with demographic changes, more widely accepted bariatric surgery, and aesthetic preferences for taut silhouettes. Minimally invasive options have expanded considerably, encompassing energy-based devices (EBDs) and regenerative injectables. Currently, preoperative decision-making relies predominantly on subjective clinical assessment. Despite its practicality, the pinch test overestimates the amount of body fat, and lacks the ability to distinguish tissue composition and assess the muscle compartment which may result in a misdiagnosis.^1^ Standard ultrasound may underestimate the thickness of subcutaneous adipose tissue (SAT) by 30% to 40% due to tissue compression in the absence of the stand-off technique.^2-4^

Two-dimensional (2D) surface classification, based solely on clinical inspection of SAT level provides no guidance as to the level of tissue composition.^5,6^ This may lead to the liposuction of low SAT positive arms or brachioplasty of sarcopenic individuals. Many of the complications of brachioplasty originate from suboptimal preoperative planning as opposed to the actual surgical technique.^7,8^

In this context, the era of empirical assessment of the arm must give way to a paradigm of precision medicine. MAALS was developed to address this gap by shifting from qualitative description to quantitative biometry. MAALS integrates high-resolution ultrasound biometry with a cylindrical geometric model, transforming linear measurements of superficial adipose tissue thickness and triceps muscle thickness into dimensionless compositional indices that objectively describe the brachial architecture.^2,9^

The aim of this manuscript is to formally define the theoretical principles, the standardized ultrasound protocol and the phenotypic classification scheme of the MAALS system, outlining a preliminary conceptual diagnostic-therapeutic algorithm for brachial laxity. No prospective clinical data are presented, but preliminary anatomically rational thresholds for adipose saturation (%SAT) and muscle filling ratio (MFR) are proposed to guide future validation studies, improve confidence in the selection of therapies, and facilitate integration with advanced technologies, including artificial intelligence.

## FUNDAMENTAL ANATOMY AND AGE-RELATED PATHOPHYSIOLOGY

### Stratified Tissue Architecture

The brachial region has multiple compartments which rely on the balance of the dermo-adipose layer and the muscle below for its aesthetic appeal. The outer layer of skin is the epidermis; beneath it lies the dermis, which is composed of papillary and reticular layers and is approximately 1-2 mm thick.^10,11^ The process of ageing causes a decrease in the stability of the dermo-epidermal junction due to the flattening of the junction.^12^

Immediately beneath the dermis lies the subcutaneous adipose tissue (SAT), organized by the superficial fascial system (SFS). The SFS is a fibroelastic lamina with an average thickness of 0.40-0.53 mm in the arm, more prominent posteriorly and richer in elastin than the deep brachial fascia. It encapsulates the SAT, provides a sliding plane and houses the subcutaneous vascular plexus and initial lymphatic capillaries, structures of particular importance for the safety of excisional procedures.^9^ Beneath the SFS, a deep adipose layer may be present, generally modest in the brachial region.^9,13^

The deep brachial fascia, thicker posteriorly (approximately 0.81 ± 0.20 mm) than the anterior portion (0.61 ± 0.11 mm), circumscribes the muscular compartment, dominated by the triceps brachii muscle, the primary extensor, and determinant of the limb's structural volume.^9,14^ Atrophy of the triceps is a direct cause of loss of support, with a reduction in the muscle's contribution to the overall arm circumference and a consequent decrease in the muscle filling ratio (MFR), one of the key parameters of MAALS.^15,16^ The main neurovascular structures—including the basilic vein and the medial brachial cutaneous nerve—run close to the deep fascia and the SFS, where they are at risk in the event of excessively deep dissection or aggressive liposuction with critical SAT.

### Molecular Pathogenesis of Laxity and Volume Loss

Arm ageing results from intrinsic ageing and photoageing, leading to reduced skin tensile strength and volume loss in adipose and muscle compartments. At the dermal level, the extracellular matrix undergoes degradation and a reduced capacity for repair. Dermal fibroblasts enter replicative senescence, with a loss of approximately 50% of their proliferative potential by the eighth decade of life and the acquisition of a senescence-associated secretory phenotype.^10,17,18^ The latter is characterized by hypersecretion of proinflammatory cytokines (IL-1α and IL-6), proteolytic enzymes and reactive oxygen species, which create a catabolic microenvironment.^17,19,20^

This catabolic state establishes an imbalance between matrix metalloproteinases (MMPs) and their inhibitors, accelerated by ultraviolet irradiation through induction of MMP-1, MMP-3, and MMP-9, leading to fragmentation of Types I and III collagen fibrils and impaired fibroblast mechanotransduction.^10-12,21^ With age, dermal collagen content decreases by over 50%, the elastic network undergoes elastotic degeneration, and glycosaminoglycan loss reduces tissue turgor and water content.^20,22^

In adipose tissue, ageing promotes lipoatrophy through adipocyte apoptosis and reduced preadipocyte differentiation, a process exacerbated by postmenopausal hormonal changes.^11^ The resulting loss of subcutaneous volume unmasks the underlying muscle atrophy characteristic of age-related sarcopenia—defined as a skeletal muscle index <7.0 kg/m^2^ in men and <5.5 kg/m^2^ in women—with progressive reduction in strength and function.^15,16,23^ The molecular substrate involves altered protein turnover, intramuscular lipid accumulation (myostatosis), and replacement of type II fibers by connective and adipose tissue^23-25^. The resulting structural collapse of the skin-adipose envelope amplifies visible ptosis and renders unselected excisional procedures counterproductive.

## CRITICAL ANALYSIS OF EXISTING CLASSIFICATION SYSTEMS

### Descriptive and Morphological Systems

Traditional classifications of brachial laxity are based primarily on visual inspection and tactile assessment. The Investigator Assessment Skin Laxity Scoring System uses a five-point scale (0-4) to assess skin laxity, consistency, and elasticity, but its inter-rater and intra-rater reliability remains moderate, with limited agreement between observers, and is insufficient for truly accurate therapeutic triage.^6^ The IBSA Inner Upper Arm Laxity Scale, developed by Cassuto et al,^6^ improves standardization through reference images but remains a 2D assessment of the skin surface, lacking information on the underlying volumetric composition. The El Khatib classification, one of the most widely used in clinical practice, combines an assessment of skin excess and adiposity to recommend different treatment options: radiofrequency (RF) for minimal cases (Grade 1), combined therapies for moderate cases (Grade 2a/b), and brachioplasty for severe cases (Grade 3). Although pragmatic, its main weakness lies in its reliance on a qualitative estimate of fat content—“mild adiposity” versus “significant”—not supported by objective measures of adipose thickness or volume distribution.^5^ Similarly, the classification by Teimourian and Malekzadeh focuses on the pattern of skin resection without including measurable tissue metrics.^26^ These systems are descriptive: they assess the degree of laxity but not tissue composition or its distribution between adipose and muscle compartments.^27^

### Fundamental Limitation: Lack of Volumetric and Compositional Data

The main limitation of conventional classifications is the lack of structural and compositional data. They quantify the degree of sagging but do not characterize its underlying composition. Thus, two patients both classified as Grade 2 according to El Khatib may present completely different anatomies: one with a 25 mm thick fibrous adipose layer and a high percentage of adipose saturation (%SAT), the other with a thin, inelastic dermis and a critical SAT of just a few millimeters. The application of the same “moderate” therapeutic algorithm—often involving liposuction—may be reasonable in the first case but disastrous in the second, causing damage to the subcutaneous plexus and increasing the risk of skin necrosis and seroma.^28^

Furthermore, current classification systems do not incorporate any parameter reflecting the state of the muscular compartment. In patients with significant triceps atrophy—whether due to age-related sarcopenia, postbariatric weight loss, or cachexia—the reduced MFR means that the muscular cylinder contributes minimally to overall limb volume. In such cases, tightening the cutaneous envelope through brachioplasty without accounting for the underlying volumetric deficit may result in an aesthetically unacceptable outcome characterized by visible bony prominences, prominent tendon contours, and a “skeletal” arm appearance. This risk, which we term the “muscle blind spot” of existing descriptive systems, cannot be identified or quantified without a compositional assessment of the muscular compartment. It is important to acknowledge, as discussed further in Phenotype 4: sarcopenic/atrophic—the structural deficit section and Rationale and clinical utility section, that this represents a theoretical risk construct at this stage, and that prospective studies are needed to confirm its clinical magnitude.^29,30^

These shortcomings highlight the need for a tool that adds a true compositional dimension to existing severity scales. MAALS was designed precisely to fill this gap, introducing two normalized dimensionless indices—%SAT and MFR—capable of objectively characterizing whether observed laxity is driven by adipose excess, dermal deficiency, or muscular deficit. From this perspective, MAALS does not replace existing descriptive scales but complements them, providing the additional data layer indispensable for truly personalized, data-driven aesthetic medicine of the arm.^5^

## THE NEW PROPOSAL MAALS PROTOCOL: LOGICAL FOUNDATIONS, STANDARDIZATION AND GEOMETRIC ALGORITHM

### Theoretical Foundations and Cylindrical Model

MAALS is based on the premise that absolute linear measurements cannot be interpreted correctly without reference to the overall scale of the body segment: a SAT of 15 mm in an arm with a circumference of 40 cm carries a very different physiological and surgical significance compared to the same thickness in an arm of 26 cm. MAALS therefore adopts a simplified cylindrical geometric model of the arm at the mid-brachial sentinel point—identified as the site of maximum ptosis^3,13^—as the reference for all biometric calculations. This geometric simplification introduces a margin of error in arms with a markedly elliptical profile, and the use of a single sentinel point—while consistent with established standardized ultrasound protocols for subcutaneous adipose tissue measurement^13,33^—represents a further simplification that does not capture regional variation in SAT thickness along the longitudinal axis of the arm. In particular, the distal third of the upper arm frequently shows greater soft-tissue envelope thickness and constitutes the primary site of ptosis and skin resection, a dimension that a single mid-brachial measurement may underestimate. These are acknowledged limitations of the current protocol, to be addressed in the prospective validation phase (see Methodological limitations and validation prospects section and Future directions section). The arm circumference (C) at this site yields the total brachial radius (Rtot = C/2π), which is conceptually divided into two echoically measurable components: the superficial dermo-adipose layer (SAT, measured from the epidermal entry echo to the deep brachial fascia, encompassing the full dermo-adipose envelope; the SFS, visible as a thin hyperechoic band within the SAT layer, acts as an internal organizing plane but does not constitute the distal measurement boundary) and the deep structural core (M, from the deep brachial fascia to the humeral cortex). Normalizing SAT and M against Rtot produces the two dimensionless indices of MAALS: the adipose saturation percentage (%SAT) and the muscle filling ratio (MFR).^13^ Although MRI remains the gold standard for softtissue compositional quantification,^31,32^ stand-off HFUS offers a substantially superior accessibility-to-accuracy ratio for outpatient clinical use, making it the preferred measurement tool within the MAALS framework.

### Standardized Ultrasonic Biometry: Overcoming Compression Bias

**Figure 1.**
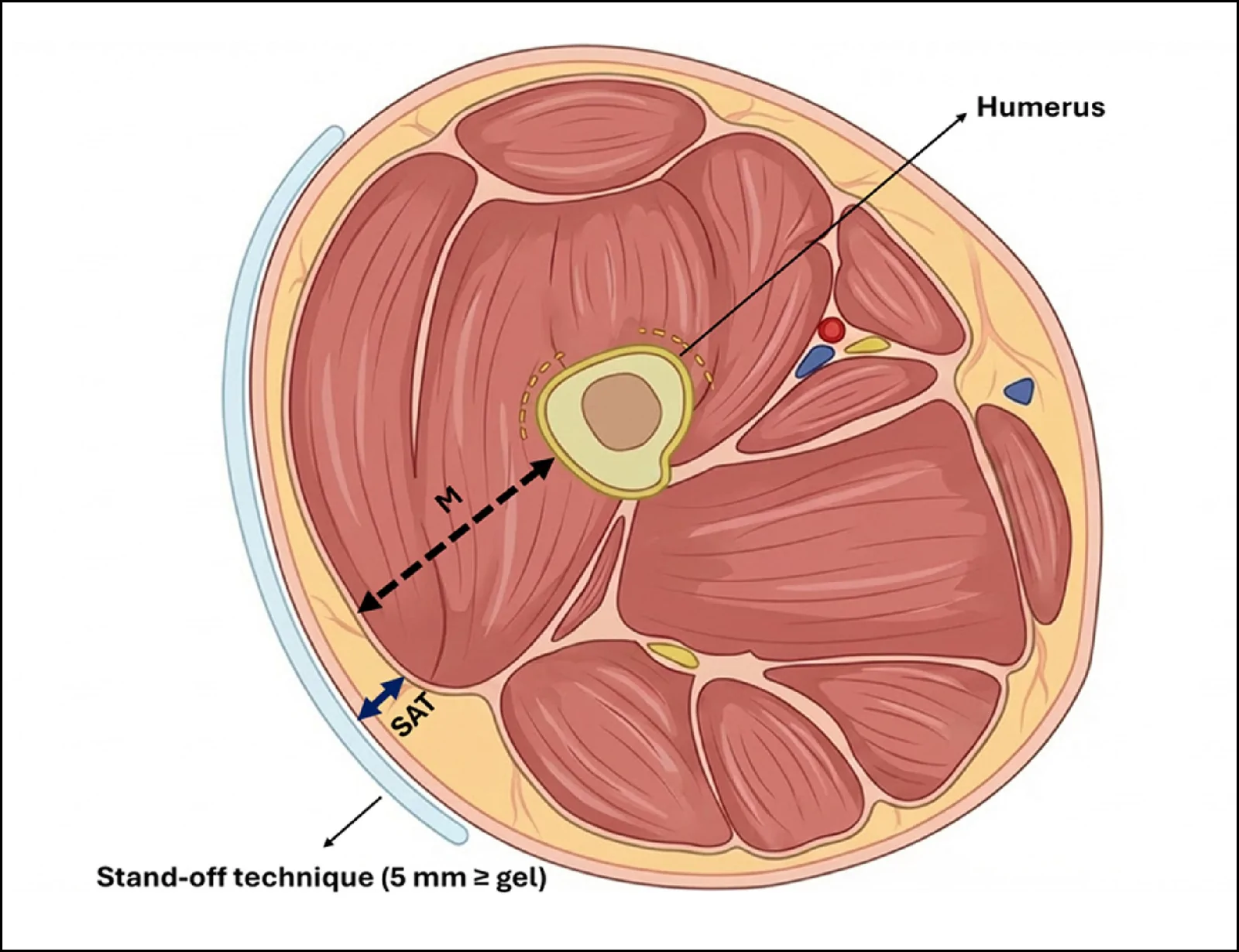
Schematic cross-section of the human arm at the mid-third of the humerus, showing the tissue layers relevant to the MAALS protocol. From the outside inwards: dermis (outer oval outline), subcutaneous adipose tissue (SAT, yellow-orange peripheral layer), superficial fascial system (SFS, golden dotted line—acting as an internal organizing plane within the SAT compartment), muscle compartment dominated by the triceps brachii posteriorly (red), humerus with periosteum and cortical bone (center). On the medial side, the main neurovascular structures are highlighted: brachial artery (red), basilic vein (blue), and medial brachial cutaneous nerve (yellow). The outer blue arc represents the ultrasound gel cushion (≥5 mm) used in the stand-off technique, necessary to eliminate compression bias in high-frequency ultrasound (HFUS) measurements. The bidirectional arrow SAT indicates the thickness of the superficial adipose tissue measured from the epidermal entry echo to the deep brachial fascia (the distal measurement boundary); the dashed arrow M indicates the thickness of the triceps muscle compartment from the deep brachial fascia to the humeral cortex; the dashed arrow Rtot indicates the total brachial radius (Rtot = C/2π, where C is the arm circumference at the sentinel point). These two parameters, normalized to Rtot, derive the two dimensionless indices of the MAALS system: the adipose saturation percentage (%SAT = SAT/Rtot × 100) and the muscle filling ratio (MFR = M/Rtot). During the preparation of this work, the authors used Figurelabs.ai to generate this schematic anatomical cross-section for illustrative purposes. After using this tool, the authors reviewed and edited the content as needed and take full responsibility for the content of the publication.

MAALS involves the use of high-frequency linear transducers (HFUS, 18-22 MHz), which offer an axial resolution of less than 80 µm, sufficient to identify the dermo-epidermal junction, the SFS plane, the deep brachial fascia, and the humeral cortex.^2,33^ To minimize compression bias—which can artificially reduce SAT thickness by more than 30% to 40%^3,4^—a no-touch stand-off technique is employed: a generous layer of ultrasound gel (≥5 mm) is interposed between the probe and the skin, allowing the transducer to float without direct contact and avoiding mechanical deformation of the compressible adipose tissue.^2,3,34^ The standardised protocol is further illustrated in Video. A schematic representation of the brachial tissue layers, SAT and M measurements, and the stand-off gel pad is illustrated in *Figure 1*.

The following operative sequence summarizes the standardized MAALS biometric procedure: Step 1. Patient positioning: Semi-recumbent at 45°, arm abducted at 90°, elbow in full extension (0° of flexion), forearm in neutral supination. This position relaxes the triceps brachii and standardizes soft-tissue tension along the posterior compartment, preventing the dimensional changes in the distal arm that occur with elbow flexion. Step 2. Sentinel point identification and circumference measurement: The mid-brachial sentinel point is identified as the midpoint between the anterior axillary fold and the tip of the olecranon, measured along the posterior arm surface with the arm at 90° abduction. Arm circumference (C) is measured at this level with a nonelastic tape measure applied without tension; Rtot is calculated as C/2π. Step 3. Equipment preparation: A high-frequency linear transducer (18-22 MHz) is connected and frequency set to maximize axial resolution (≤80 µm). Gain and depth are adjusted to display the full soft-tissue envelope from the epidermal surface to the humeral cortex. Step 4. Stand-off gel pad application: A generous layer of coupling gel (≥5 mm depth, verified by on-screen ruler before each measurement) is applied to the skin at the sentinel point. The gel must form a continuous, bubble-free cushion before transducer placement. Step 5. No-touch transducer technique: The transducer is placed on the gel layer in a transverse orientation, perpendicular to the skin surface, and allowed to float without exerting any direct pressure. The operator's hand supports the transducer weight only; mechanical contact with the skin is to be strictly avoided to prevent compression bias. Step 6. Landmark identification: In the transverse plane, four hyperechoic landmarks are sequentially identified: (i) the epidermal entry echo (bright superficial line); (ii) the SFS plane (thin echogenic band within the SAT layer); (iii) the deep brachial fascia (dense hyperechoic line at the SAT–muscle interface); and (iv) the humeral cortex (curved hyperechoic arc with posterior acoustic shadowing). Step 7. SAT and M measurement: SAT thickness is measured perpendicularly from the epidermal entry echo to the deep brachial fascia plane using the system's electronic calipers. M (triceps muscle thickness) is measured from the deep brachial fascia to the humeral cortex. Both measurements are repeated three times consecutively without repositioning the transducer; the mean of the three readings is used for index calculation. Step 8. Index calculation: %SAT = (SAT/Rtot) × 100; MFR = M/Rtot. The resulting indices are entered into *Table 1* for phenotypic assignment.

**Table 1.** Phenotypic Classification of the MAALS (Melfa-Anastasia Arm Laxity System) With Diagnostic Thresholds and Preliminary Therapeutic Indications

Phenotype	Name	%SAT	SAT (mm)	MFR	Main therapeutic indication
0	Normotrophic	<20%	≤12 mm	>0.35	Prevention; no ablative or excisional procedures
1	Adipose hypertrophy	>30%	>15 mm	>0.25	Liposuction (UAL/VASER); noninvasive fat reduction
2	Mixed morphology	20%-30%	8-15 mm	0.20-0.30	LAL/RFAL; lipobrachioplasty
3	Skin ptosis—vascular risk zone	<20%	<4-5 mm	Variable	Excision brachioplasty; liposuction contraindicated
4	Sarcopenic/atrophic	Low	Negligible	<0.20	Additive/regenerative approach (PLLA, CaHA, lipofilling, HIFEM)

The percentage of superficial adipose saturation of the radius (%SAT) is calculated as %SAT = (SAT/Rtot) × 100; the muscle filling ratio (MFR) is calculated as MFR = M/Rtot, where Rtot = C/2π (C = brachial circumference at the mid-third). SAT, thickness of superficial adipose tissue measured by high-frequency ultrasound (HFUS) using the stand-off technique. In Phenotype 3, MFR is listed as variable because the defining criterion is critical SAT thickness (<4-5 mm), not the degree of muscle filling. The thresholds reported are preliminary, derived from an anatomical-rational synthesis of the available literature, and subject to future prospective validation.

### Derivation of Predictive Indices and Phenotypic Thresholds

The measured values (C, SAT, M) are converted into two dimensionless indices forming the diagnostic core of MAALS. The first, the adipose saturation percentage (%SAT), is calculated as the ratio of SAT thickness to the total arm radius, multiplied by one hundred: %SAT = (SAT/Rtot) × 100. High values indicate a predominantly adipose cylinder, whilst low values identify conditions of critical SAT, in which the subcutaneous vascular plexus and lymphatic collectors come dangerously close to the dermis.

The second index is the muscle filling ratio (MFR), calculated as the ratio of the thickness of the triceps muscle to the total radius: MFR = M/Rtot. This parameter quantifies the relative contribution of the triceps muscle to the limb's overall radius, serving as a proxy for deep structural integrity and for the risk of “skeletal” outcomes following aggressive subtraction or excision procedures.

**Figure 2.**
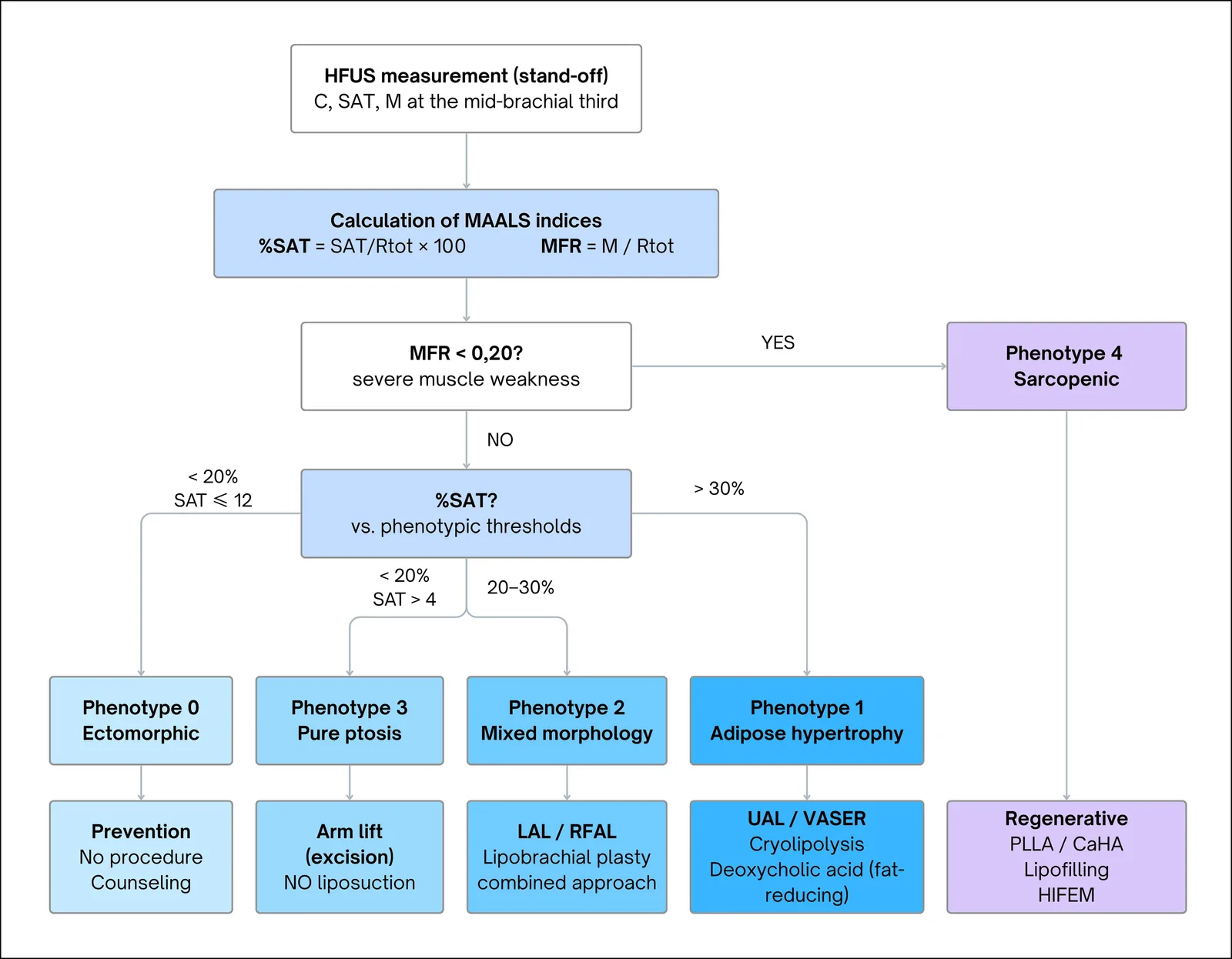
Diagnostic-therapeutic algorithm of the MAALS system (Melfa-Anastasia Arm Laxity System). The decision-making pathway begins with standardized ultrasound measurement using the stand-off technique (HFUS, 18-22 MHz) at the mid-brachial third, from which the system's two dimensionless indices are derived. The first branch evaluates the MFR: a value below 0.20 directly identifies Phenotype 4 (sarcopenic/atrophic), for which an additive and regenerative approach (PLLA, CaHA, lipofilling, and HIFEM) is indicated. In the absence of marked muscle deficit, the second branch evaluates %SAT against preliminary phenotypic thresholds: %SAT <20% with SAT ≤12 mm and MFR >0.35 points toward Phenotype 0 (normotrophic arm, prevention, no subtractive procedures); %SAT <20% with SAT <4-5 mm and clinically evident ptosis points toward Phenotype 3 (pure skin ptosis, excisional brachioplasty; liposuction strictly contraindicated due to risk of skin necrosis from critical SAT); %SAT 20% to 30% points toward Phenotype 2 (mixed morphology, LAL/RFAL, lipobrachioplasty); %SAT >30% toward Phenotype 1 (adipose hypertrophy, UAL/VASER, noninvasive lipolysis). All thresholds are preliminary and subject to future prospective validation. LAL, laser-assisted lipolysis; RFAL, radiofrequency-assisted liposuction; UAL, ultrasound-assisted liposuction; PLLA, poly-L-lactic acid; CaHA, calcium hydroxyapatite; HIFEM, high-intensity focused electromagnetic; HFUS, high-frequency ultrasound; MFR, muscle filling ratio.

Based on anatomical evidence and clinical data in the literature, MAALS proposes preliminary thresholds for phenotypic stratification, to be considered provisional and subject to future prospective validation. A %SAT greater than 30% (Phenotype 1) is indicative of predominant adipose hypertrophy, with SAT generally greater than 15 mm and adequate safety conditions for liposuction. A %SAT between 20% and 30% (Phenotype 2) represents a mixed morphology, with medium-thickness SAT (approximately 8-15 mm) and frequent septal and cutaneous laxity. A %SAT below 20% (Phenotype 3) represents the critical threshold, with dangerously thin SAT (< 4-5 mm), and constitutes a “vascular risk zone” in which liposuction is strongly discouraged. Finally, an MFR of less than 0.20 (Phenotype 4) indicates a marked deficit in muscle filling, constituting the sarcopenic/atrophic arm in which isolated excisional procedures are inherently risky. Phenotype 0 (normotrophic) is defined by %SAT below 20%, SAT not exceeding 12 mm and MFR above 0.35, indicative of an adequately represented muscle compartment in the absence of clinically relevant adipose hypertrophy. By combining the %SAT and MFR thresholds, MAALS defines a total of five clinical phenotypes (0-4), summarized in *Table 1* and described in detail in the following section. The diagnostic-therapeutic algorithm derived from the phenotypic classification is schematically represented in *Figure 2*.

## COMPLETE PHENOTYPIC STRATIFICATION AND THERAPEUTIC CORRELATIONS

### Phenotype 0: The Physiological (Normotrophic) Arm

Phenotype 0 identifies the normotrophic physiological arm: brachial circumference typically less than 26 cm, %SAT less than 20% with SAT ≤ 12 mm and MFR greater than 0.35. On HFUS ultrasound, the profile is that of a structurally well-supported arm: SAT of reduced thickness and homogeneous sublayer distribution, well-oriented dermal bands and a hyperechoic triceps muscle with preserved myofibrillar architecture. No signs of septal thinning or irregularities of the deep fascial plane are observed.

**Figure 3.**
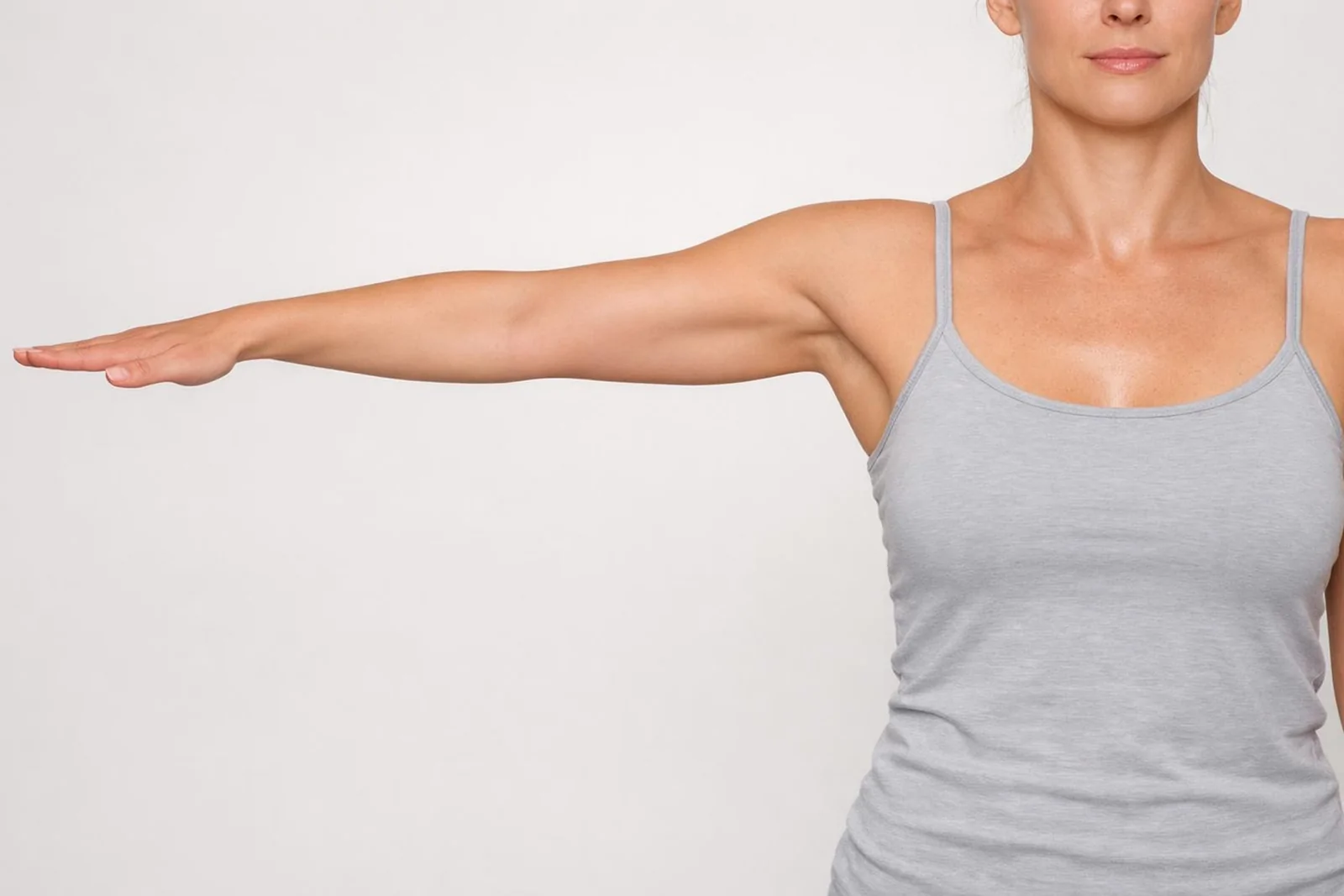
Representative image of MAALS Phenotype 0—normotrophic arm. Representative subject: healthy young adult female. The arm displays a well-defined contour with no visible ptosis, adequate subcutaneous adipose tissue distribution (%SAT <20%, SAT ≤12 mm), and preserved muscular filling ratio (MFR >0.35). The triceps profile is firm and well-supported, with no redundant skin envelope. This phenotype requires no aesthetic intervention; counseling on lifestyle maintenance and prevention represents the only indicated approach. Anterior view, arm abducted at 90°. During the preparation of this work, the authors used Artlist.io to generate this representative phenotypic image for educational illustration purposes. After using this tool, the authors reviewed and edited the content as needed and take full responsibility for the content of the publication. This image does not represent a real patient. MFR, muscle filling ratio.

Clinically, the main diagnostic risk is confusing dynamic pseudoptosis with true primary laxity. The pinch test systematically overestimates the fat content in these arms, leading the clinician to hypothesize a lipodystrophic component that does not exist. The apparent movement of the skin during arm abduction is an expression of the skin's normal viscoelastic behavior, not of treatable tissue excess.^5,26^ A representative clinical presentation of Phenotype 0 is illustrated in *Figure 3*.

The therapeutic implications are straightforward: any subtractive or excisional procedure is absolutely contraindicated. With a low SAT, fat removal would expose the subcutaneous vascular plexus to an unacceptable risk of ischemic and lymphatic damage.^5^ Management focuses on prevention and the maintenance of tissue quality: topical retinoids and antioxidants to preserve skin integrity, nonablative fractional laser (1540-1550 nm) to stimulate collagen remodeling without altering the volumetric structure, and high-intensity focused electromagnetic (HIFEM) devices for muscle tone without reducing adipose volume.^35^ The primary objective remains providing the patient with informed counseling, aimed at avoiding unnecessary and potentially harmful treatments in an arm that does not require corrective intervention.

### Phenotype 1: True Adipose Hypertrophy

**Figure 4.**
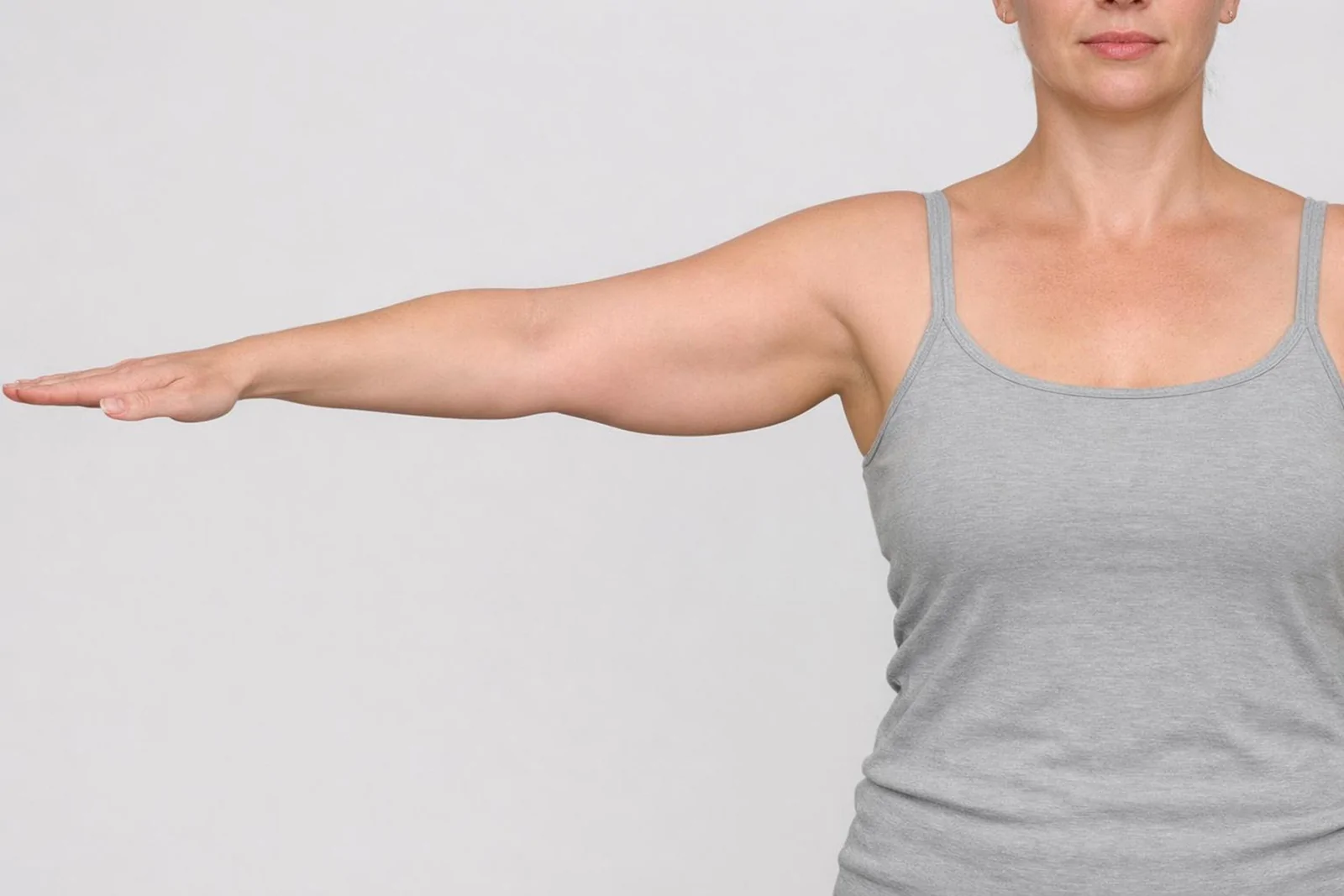
Representative image of MAALS Phenotype 1—Adipose Hypertrophy. Representative subject: healthy young adult female. The arm displays a localized adipose accumulation in the proximal third, producing a focal convexity at the axillary-brachial junction, with %SAT >30% and SAT generally exceeding 15 mm at the sentinel point. The distal arm contour remains well-defined and the skin envelope is elastic, with a preserved MFR >0.25. This presentation is amenable to subtractive approaches—ultrasound-assisted liposuction (UAL/VASER) or noninvasive adipolysis—with predictable skin retraction given the preserved dermal quality. Anterior view, arm abducted at 90°. During the preparation of this work, the authors used Artlist.io to generate this representative phenotypic image for educational illustration purposes. After using this tool, the authors reviewed and edited the content as needed and take full responsibility for the content of the publication. This image does not represent a real patient. MFR, muscle filling ratio.

Phenotype 1 is characterized by a SAT% greater than 30%—with SAT frequently exceeding 15 mm—and an MFR usually greater than 0.25, indicating a muscle compartment that is still structurally competent. It is prevalent in younger individuals and in patients with a familial pattern of upper limb lipodystrophy.^36^ On high-frequency ultrasound (HFUS), SAT appears as a thick, homogeneous hypoechoic layer, with distinct connective septa and a well-defined muscle fascia beneath. The humeral cortex is easily identifiable, and the triceps muscle shows preserved architecture.^13,37^ The clinical appearance is that of a cylindrical, compact arm, with loss of definition of the muscle borders due to excess fat, in the absence of true primary skin laxity. A representative clinical presentation of Phenotype 1 is illustrated in *Figure 4*.

The treatment algorithm prioritizes selective volume reduction, within a context of abundant subcutaneous fat that ensures an adequate vascular safety margin.^5^ The gold standard for this phenotype is third-generation ultrasound-assisted liposuction (UAL/VASER; Solta Medical, Bothell, WA, USA), which utilizes low-energy pulsed ultrasonic cavitation to selectively emulsify adipocytes whilst preserving the neurovascular stromal network.^37,38^ Compared to suction-assisted liposuction, UAL results in less bleeding and greater postprocedural skin retraction, estimated at up to a 25% reduction in circumference.^5,37^ In milder forms of adipose hypertrophy, or when the patient refuses surgery, noninvasive adipolysis technologies may be considered: cryolipolysis (apoptosis via controlled crystallization of adipocytes) or deoxycholic acid injections (focal chemical adipocytolysis).^39^ Brachioplasty is generally not indicated in this phenotype: performing skin resection on a flap that is still volumetrically redundant generates excessive tension on the suture, with a risk of scar widening and migration.^5^

### Phenotype 2: Mixed Morphology—The Therapeutic Crossroads

**Figure 5.**
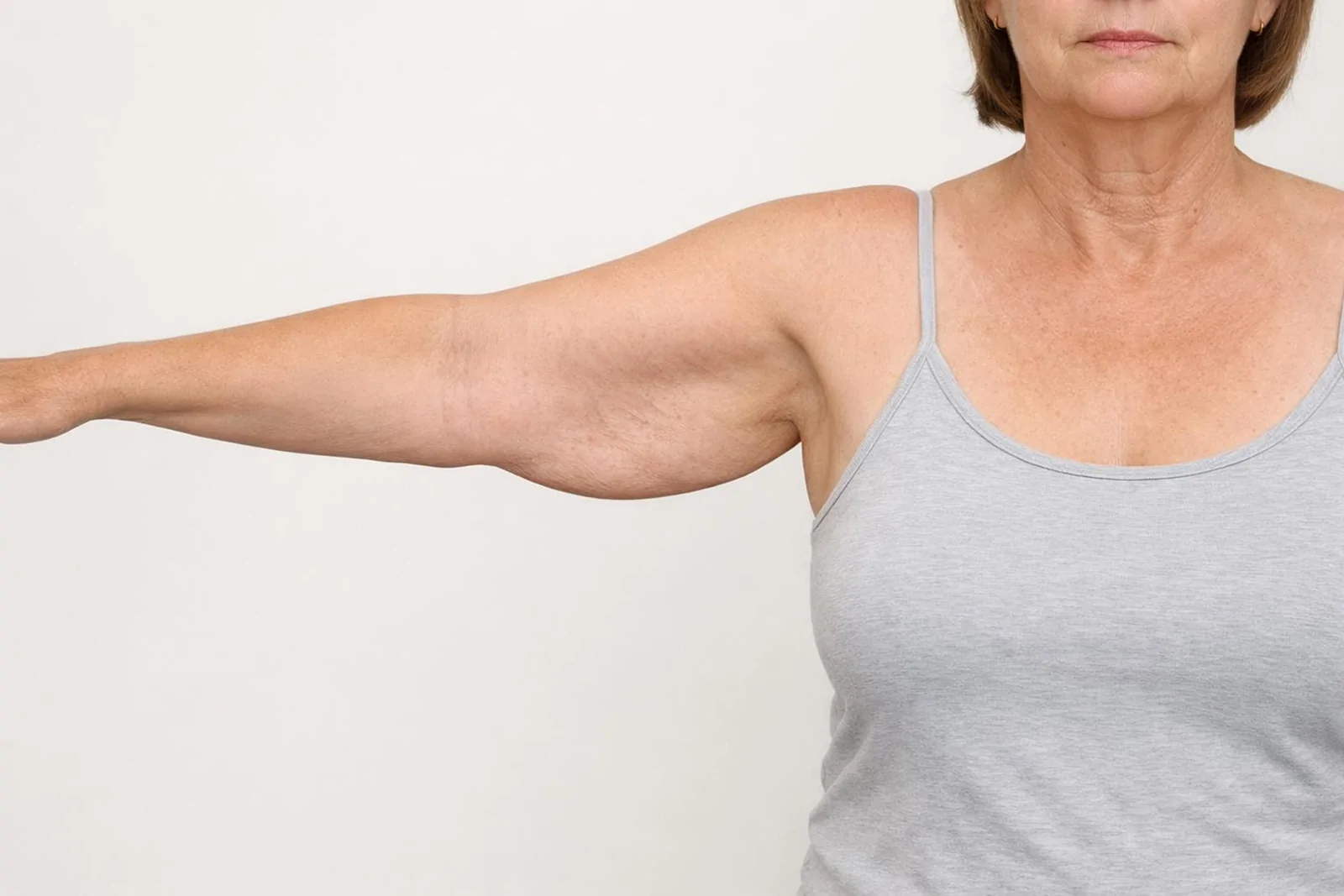
Representative image of MAALS Phenotype 2—mixed morphology. Representative subject: healthy mature adult female. The arm displays a combination of moderate subcutaneous adipose excess (%SAT 20%-30%, SAT 8-15 mm at the sentinel point) and a dermal envelope with visibly reduced elasticity, characterized by skin redundancy along the postero-inferior surface, surface wrinkling, and early ptosis. The MFR remains within the preserved range (0.20-0.30), indicating adequate muscular support. The coexistence of adipose excess and compromised skin quality defines the mixed tissue phenotype and requires a combined therapeutic approach—laser-assisted lipolysis (LAL) or radiofrequency-assisted lipolysis (RFAL) for volume reduction and simultaneous dermal tightening, with lipobrachioplasty reserved for cases with more advanced skin redundancy. Anterior view, arm abducted at 90°. During the preparation of this work, the authors used Artlist.io to generate this representative phenotypic image for educational illustration purposes. After using this tool, the authors reviewed and edited the content as needed and take full responsibility for the content of the publication. This image does not represent a real patient. MFR, muscle filling ratio.

Phenotype 2, with %SAT between 20% and 30% and SAT typically between 8 and 15 mm, is the most heterogeneous and clinically common.^5^ The MFR value is variable, but generally lies between 0.20 and 0.30. On HFUS ultrasound, the adipose layer is of moderate thickness and may show the first signs of skin thinning, with reduced dermal echogenicity; the SFS sometimes appears undulating or partially detached, indicating a loss of tension in the SFS.^37^ The clinical and echoic picture is that of an arm in which visible excess fat coexists with posterior skin laxity: this reflects both volume excess and dermal laxity, but a dual condition requiring a synergistic therapeutic approach. A representative clinical presentation of Phenotype 2 is illustrated in *Figure 5*.

The therapeutic approach for Phenotype 2 involves a simultaneous reduction in volume and tissue firming. Laser-assisted lipolysis (LAL) using a 1470 nm diode fiber (eg, Endolift systems; Eufoton S.r.l., Trieste, Italy) enables dual-wavelength absorption—on fat for lipolysis and on water for coagulation—which produces immediate collagen contraction and progressive neocollagenesis, reducing both volume and laxity in a single session.^40,41^ Alternatively, RF-assisted platforms [eg, QuantumRF (InMode Ltd., Yokneam, Israel) or BodyTite (InMode Ltd., Yokneam, Israel) or ThermiTight (ThermiAesthetics, Southlake, TX, USA)] deliver volumetric heating to the subcutaneous plane, inducing collagen denaturation and subsequent reorganization, with an immediate firming effect and long-term fibroblast stimulation, usually in combination with conservative liposuction.^28,42^ In cases where there is significant excess skin and the adipose component remains within safe limits, lipobrachioplasty—which combines conservative liposuction to reduce the weight of the flap and lower tension on the suture, followed by limited skin excision—offers an optimal morphological result whilst minimizing the risk of scarring.^40^ The choice between these options should be guided by quantitative MAALS values and the patient's preferences, not by purely empirical criteria.

### Phenotype 3: Pure Cutaneous Ptosis—The Vascular Risk Zone

**Figure 6.**
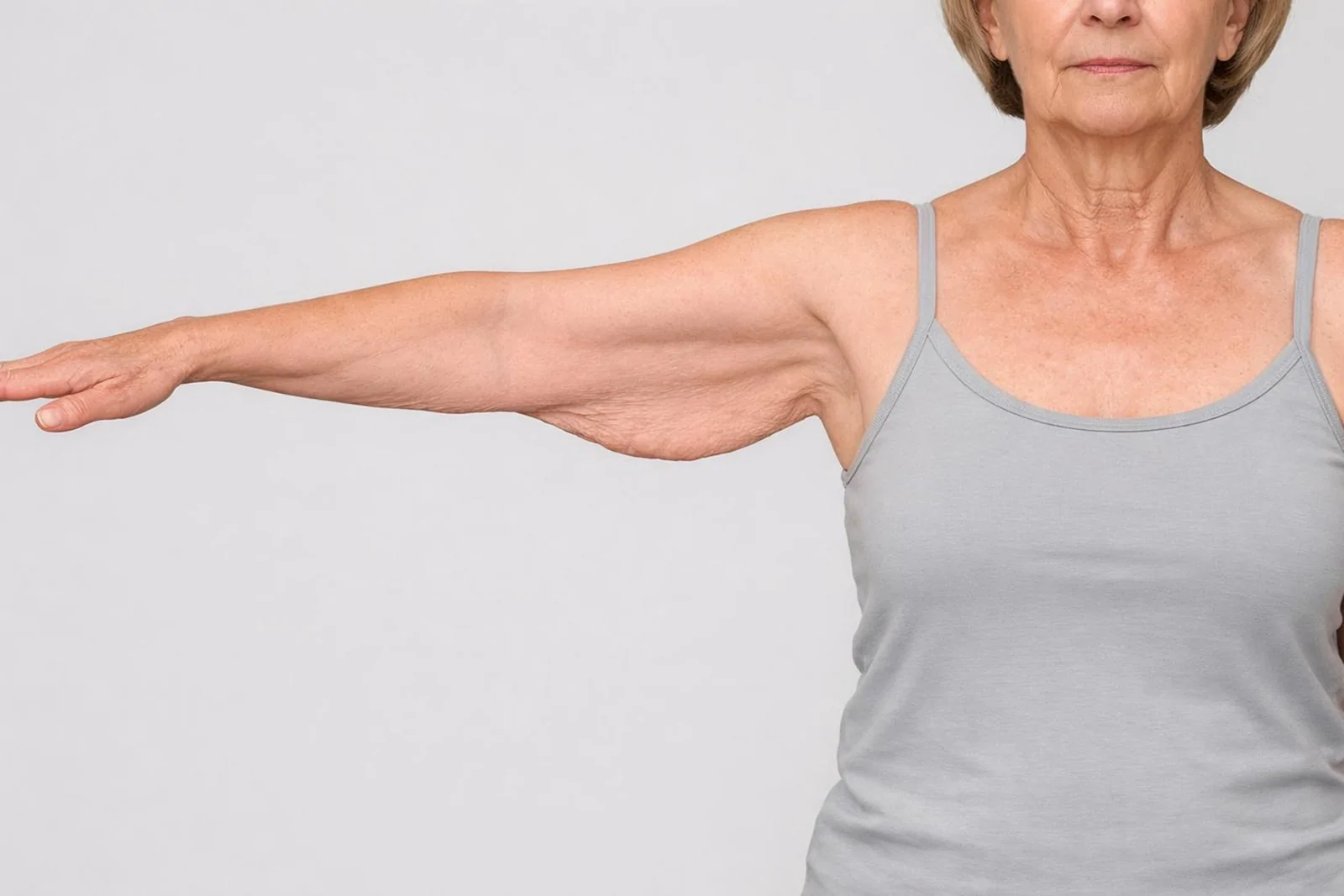
Representative image of MAALS Phenotype 3—pure cutaneous ptosis. representative subject: healthy older adult female. The arm displays marked skin redundancy along the postero-inferior surface with visible dependent ptosis of 2-5 cm, in the absence of significant subcutaneous adipose excess (%SAT <20%, SAT <4-5 mm at the sentinel point). The skin envelope is thin, inelastic, and shows surface wrinkling consistent with advanced dermal ageing; the underlying muscular profile remains appreciable through the atrophic subcutaneous layer. The MFR is variable. This phenotype defines the critical vascular risk zone of the MAALS system: the proximity of the subcutaneous vascular plexus and lymphatic collectors to the dermis renders liposuction absolutely contraindicated. Excisional brachioplasty with the Lockwood SFS technique represents the treatment of choice. Anterior view, arm abducted at 90°. During the preparation of this work, the authors used Artlist.io to generate this representative phenotypic image for educational illustration purposes. After using this tool, the authors reviewed and edited the content as needed and take full responsibility for the content of the publication. This image does not represent a real patient. MFR, muscle filling ratio.

Phenotype 3 is defined by a %SAT of less than 20%, with SAT generally less than 4-5 mm; the MFR value is variable but does not constitute the distinguishing parameter for this phenotype.^5^ The high-frequency ultrasound (HFUS) picture is characteristic and unambiguous: the adipose layer is thin or almost absent, the dermis appears hyperechoic and reduced in thickness, and the space between the deep fascia and the dermis is occupied by loose, hypoechoic areolar tissue, indicative of a virtually depleted panniculus.^37^ The clinical pattern of the so-called “empty sac deformity” or pure “bat wing” is recognized, in which the skin folds and sags in the absence of any real underlying excess fat. The pinch test in this phenotype systematically overestimates adiposity, primarily collecting skin and areolar tissue rather than actual fat, and its use as a guide to therapeutic decision-making can lead to dramatic errors.^5^ A representative clinical presentation of Phenotype 3 is illustrated in *Figure 6*.

Liposuction is contraindicated in this phenotype: with SAT below 4-5 mm, the vascular and lymphatic plexuses lie in proximity to the dermis, increasing the likelihood of skin necrosis, contour deformities, and chronic seroma formation.^29,43,44^ The definitive and elective treatment for Phenotype 3 is excisional brachioplasty, which must be performed with the utmost care to preserve the thin residual panniculus, in order to ensure cutaneous perfusion of the flap. The suspension technique to the SFS and axillary fascia, described by Lockwood, is essential for achieving a stable lift and reducing downward migration of the scar over time.^44,45^ In carefully selected patients with residual dermal elasticity and moderate ptosis, RF-based approaches may offer adjunctive tissue toning and subdermal retraction: RF-assisted lipolysis with continuous external thermal monitoring represents the preferred option when SAT approaches the critical threshold,^43^ while fractional RF microneedling may complement tissue remodeling through neocollagenesis in patients with slightly higher SAT values^46^; in both cases, intraoperative HFUS monitoring of SAT thickness is mandatory. In patients who decline surgery, nonsurgical approaches—including MFU-V, RF microneedling and hyperdiluted calcium hydroxyapatite (CaHA)—may improve skin quality but offer limited morphological correction; realistic expectations must be discussed with the patient.

### Phenotype 4: Sarcopenic/Atrophic—The Structural Deficit

**Figure 7.**
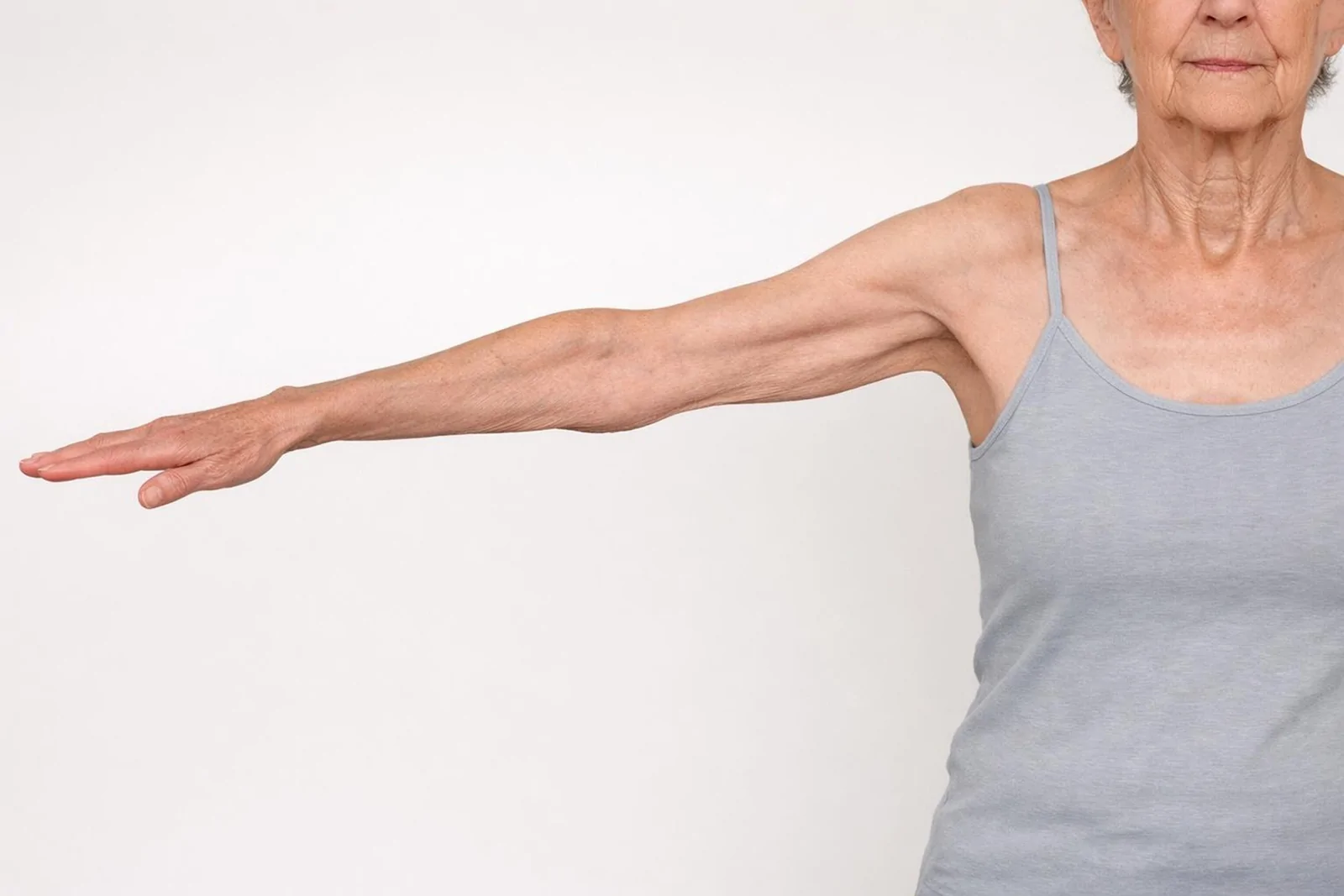
Representative image of MAALS Phenotype 4—sarcopenic/atrophic arm. Representative subject: healthy elderly adult female. The arm displays a markedly reduced total radius (Rtot), with negligible subcutaneous adipose tissue (SAT negligible, %SAT low) and a severely compromised muscle filling ratio (MFR <0.20). Bony prominences are clearly visible at the humeral shaft, tendons are apparent through the skin surface, and the skin envelope is thin, inelastic, and intimately adherent to the underlying atrophic muscular and connective tissue. This presentation reflects sarcopenic structural collapse and represents a paradigm inversion in therapeutic strategy: all subtractive procedures are contraindicated. The indicated approach is exclusively additive and regenerative, prioritizing volume restoration (PLLA, hyperdiluted CaHA, autologous lipofilling) and muscular stimulation (HIFEM). A staged two-session approach is recommended when brachioplasty is clinically unavoidable. Anterior view, arm abducted at 90°. During the preparation of this work, the authors used Artlist.io to generate this representative phenotypic image for educational illustration purposes. After using this tool, the authors reviewed and edited the content as needed and take full responsibility for the content of the publication. This image does not represent a real patient. MFR, muscle filling ratio.

Phenotype 4 is identified by an MFR of less than 0.20, typically accompanied by a low %SAT, and is predominantly found in elderly individuals, cachectic patients, or in some postbariatric patients with massive weight loss.^16^ On HFUS, the belly of the triceps muscle appears thin and shows increased intramuscular echogenicity, indicative of intramyofibrillar lipid infiltration (myostatosis); the SAT layer is negligible and the total limb radius (Rtot) is reduced compared to the previous phenotypes.^37,42,47^ The clinical presentation is that of a skeletal-looking arm: bony prominences are evident, tendons are visible and the skin, lacking muscular and adipose support, appears thin and wrinkled, with ptosis reflecting profound structural collapse rather than tissue excess. A representative clinical presentation of Phenotype 4 is illustrated in *Figure 7*. The visible bony prominences, tendon definition, and complete absence of subcutaneous volume represent the hallmark features of sarcopenic structural collapse.

This phenotype requires a shift in therapeutic approach, reversing the subtractive logic in favor of an additive and regenerative approach. The restoration of tissue volume and quality is the primary objective: poly-L-lactic acid (PLLA) and hyperdiluted CaHA are the biostimulatory agents of choice, inducing progressive neocollagenesis and fibroblast activation, respectively^48-55^ Autologous mini-lipofilling with nano-fat or stromal vascular fraction contributes to both skin quality and subtle volumetric restoration^56-59^. HIFEM devices can selectively hypertrophy the remaining viable muscle fibers, progressively improving MFR^47,60-63^. When brachioplasty is clinically indicated in Phenotype 4 patients, the sequencing of regenerative and resective steps is critical. A staged two-session approach is conceptually preferable in most cases: the first session prioritizes volumetric restoration through biostimulatory agents (PLLA, hyperdiluted CaHA), autologous lipofilling onto the muscle surface, and initiation of an HIFEM programme; the second session, typically planned at 3-6 months following reassessment of MFR and SAT values, addresses residual skin excess through conservative brachioplasty. This sequencing ensures that skin excision is performed against an improved structural base, reduces the risk of “skeletal” contour outcomes, and allows the regenerated panniculus to serve as a vascular recipient bed for fat grafts applied at the time of excision. In cases where simultaneous intervention is unavoidable, fat grafting onto the muscle surface must precede skin resection within the same operative session, and skin excision must remain moderate and technically conservative.^28,45^

## INTEGRATION WITH MODERN THERAPEUTIC TECHNOLOGIES

### Energy-Based Devices for Remodeling and Firming

In the context of MAALS, EBDs are not a separate category, but are positioned along the phenotypic continuum as tools that can be modulated according to %SAT and MFR. In Phenotypes 1 and 2, characterized by a still significant adipose component and a structurally recoverable dermal layer, technologies focused on volume reduction and skin tightening are clinically the most appropriate.

Visualized microfocused ultrasound (MFU-V, eg, Ultherapy; Merz Aesthetics, Raleigh, NC, USA) allows thermal coagulation points to be focused at predetermined depths (4.5 mm and beyond), stimulating controlled neocollagenesis and improving skin firmness in cases of mild-to-moderate laxity typical of the upper range of Phenotype 2. In clinical trials, this modality has demonstrated significant increases in Global Aesthetic Improvement Scale scores at 90 and 180 days, with a favorable safety profile.^50,51^ RF technologies include monopolar systems for deep volumetric heating (such as Thermage; Solta Medical, Bothell, WA, USA) and fractional platforms for controlled ablation and coagulation in the dermis and superficial subcutaneous tissue (such as Morpheus8; InMode Ltd., Yokneam, Israel). Within the MAALS framework, these devices are primarily indicated for Phenotypes 1 and 2, where they can help improve the quality of the dermal matrix and induce moderate skin tightening, complementing conservative ablative techniques when the %SAT permits.^62,63^

Nonablative fractional lasers (1540-1550 nm) can be applied across all phenotypes except advanced Phenotype 3 to optimize skin surface quality without altering volumetric structure^35^; in Phenotypes 1 and 2, combination with a 1064 nm Nd:YAG laser may contribute complementary lipolysis and modest tightening.^64,65^

### Surgical Improvements Guided by MAALS

The quantitative parameters of MAALS provide additional information for surgical planning to plan brachioplasty and combined procedures more precisely. In Phenotype 2, the ultrasound-defined SAT thickness allows quantification of the amount of adipose tissue that can be safely removed from the flap prior to skin resection, minimizing the risk of vascular compromise and contour irregularities.^9^ In phenotypes with a high %SAT, MAALS values may justify a wider elliptical resection, given that the residual adipose pad is sufficient to protect the subcutaneous plexus; conversely, in the presence of a critical %SAT (Phenotype 3), the system clearly indicates the need to preserve as much subcutaneous tissue as possible and rules out concomitant liposuction.

In Phenotype 4, the preoperatively calculated MFR deficit can be used as a reference to estimate the volumes to be restored with fat grafts or biostimulators, both when brachioplasty is unavoidable and when nonsurgical approaches are chosen. In this way, MAALS does not merely select candidates for surgery but helps to define the combined strategy of resection, subtraction, and augmentation, with the aim of achieving less redundant arms without resulting in skeletal outcomes^52,55-58^.

## DISCUSSION

### Rationale and Clinical Utility

MAALS is presented here strictly as a hypothesis-generating conceptual framework for precision medicine, not as a validated standard of care. The proposed phenotypic thresholds are derived from an anatomically rational synthesis of the peer-reviewed literature and must be considered preliminary in every respect. No prospective clinical data are presented, and the system does not yet meet the evidentiary requirements of a validated classification system. The proposed thresholds are intended to serve as a structured starting point for prospective multicenter studies designed to correlate MAALS indices with clinical outcomes—including complication rates, patient-reported outcome measures, and independent aesthetic assessments—which are explicitly outlined in Methodological limitations and validation prospects section. The treatment ramifications described for each phenotype reflect the logical application of established anatomical and physiological principles, not yet validated therapeutic algorithms. This distinction is essential when interpreting the following considerations in terms of their potential, not as confirmed evidence. The first advantage is reduced compression bias: the stand-off technique, already validated in the fields of nutrition and sports for the measurement of subcutaneous adipose tissue, offers more accurate SAT measurements than the pinch test or skinfold measurements, with high interobserver intraclass correlation coefficients (ICC) at standardized sites.^66,67^ Normalization against Rtot—derived from brachial circumference—allows the same absolute SAT thickness to be interpreted in relation to the limb's overall dimensions, overcoming the main limitation of isolated linear measurements that do not account for individual body scale.

The second distinctive advantage of MAALS over existing scales is the inclusion of the muscle compartment in the diagnostic equation. None of the current tools include a parameter to identify sarcopenic patients, rendering one of the most significant risk factors in brachial remodeling procedures invisible. The proposed %SAT and MFR thresholds are supported by anatomical evidence and literature data: the %SAT threshold of <20% as a vascular risk zone is consistent with histological studies placing the subcutaneous plexus and lymphatic collectors 1-3 mm from the dermis in conditions of thin panniculus, with documented higher rates of necrosis and seroma following aggressive liposuction associated with brachioplasty^29,43^; the MFR threshold < 0.20 for Phenotype 4 is supported by physiological reasoning regarding the relationship between muscular volume and the structural support of the cutaneous envelope after resective procedures, and by broader evidence linking sarcopenia to increased complications in body contouring surgery generally.^30,68^ We acknowledge, as raised during peer review, that direct evidence specifically correlating sarcopenia with brachioplasty outcomes is not yet available in the literature. In selected patients whose primary goal is circumferential reduction, a low MFR may not be associated with an unsatisfactory result. The purpose of MAALS Phenotype 4 is not to contraindicate brachioplasty in all sarcopenic patients, but to flag the compositional risk of “skeletal” outcomes and to prompt the surgeon to incorporate a pre-resective volumetric strategy where indicated. Prospective studies specifically evaluating MFR as a predictor of brachioplasty outcomes are needed to establish the clinical weight of this parameter.

### Methodological Limitations and Validation Prospects

The implementation of MAALS in clinical practice faces several methodological constraints. The requirement for HFUS transducers (18-22 MHz) and operators specifically trained in the stand-off technique may limit accessibility in settings lacking appropriate equipment.^67,69,70^ When the technique is correctly applied—with adequate gel pad thickness and no inadvertent probe pressure—interobserver reliability is high at standardized sites; however, inexperienced operators are susceptible to reintroducing compression bias, and a structured training pathway is therefore essential before MAALS measurements can be considered clinically reliable.^67,70^

The proposed %SAT and MFR thresholds remain preliminary and must be regarded as anatomically rational estimates rather than validated cut-offs. Prospective multicenter studies are needed to correlate MAALS indices with objective endpoints: complication rates (seroma, skin necrosis, sensory disturbance), validated patient-reported outcome measures such as the BODY-Q Arms module,^71,72^ and independent aesthetic assessments at medium and long-term follow-up. Such studies should specifically evaluate inter-operator reproducibility across multiple centers, assess threshold performance in currently underrepresented populations—including male patients and postbariatric patients with distinctive tissue profiles and elevated complication risk^73^—and determine whether sub-classification within phenotypes is warranted by the data. Furthermore, the use of a single sentinel measurement point is an acknowledged simplification: validation studies should assess whether a multisite protocol—including measurement at the distal sentinel, at the midpoint between the olecranon and the sentinel point, and at the midpoint between the axilla and the sentinel point—captures clinically important regional variation along the arm's longitudinal axis and improves phenotypic accuracy.

### Future Directions

The natural evolution of MAALS lies in its integration with advanced diagnostic technologies. In the field of artificial intelligence, the dermal, fascial and osseous interfaces visible in brachial HFUS images are well-suited candidates for automated segmentation: algorithms trained on large annotated ultrasound datasets could calculate %SAT and MFR in real time, independently of the operator, transforming MAALS from a manual measurement protocol into a standardized point-of-care diagnostic tool.^74,75^ The parallel development of shear-wave elastography (SWE)—a technique validated for superficial soft tissue characterization across multiple clinical contexts^76^—offers a complementary biomechanical dimension, combining tissue stiffness quantification in kilopascals with the compositional profile of %SAT and MFR to achieve a pretreatment assessment that is simultaneously anatomical, compositional and functional, with diagnostic potential exceeding that of morphological ultrasound alone. The standardization of a multisite measurement protocol—specifically, a three-point assessment at the mid-brachial sentinel, at the midpoint between the olecranon and the sentinel, and at the midpoint between the axilla and the sentinel—represents a concrete methodological evolution to be prospectively evaluated, as it would address the regional anatomical variability of the upper arm that a single-point protocol cannot capture. Finally, the clinical adoption of MAALS requires its incorporation into structured training programmes in aesthetic medicine and plastic surgery, fostering a culture of objective, data-driven decision-making as a prerequisite for safe and individualized brachial remodeling.

## CONCLUSIONS

The Melfa-Anastasia Arm Laxity System (MAALS) is a conceptual diagnostic-therapeutic framework that integrates high-frequency ultrasound (HFUS) biometry with a cylindrical geometric model to derive two dimensionless indices—the percentage of adipose saturation (%SAT) and the muscle filling ratio (MFR)—capable of objectively describing the brachial architecture. The resulting five-category phenotypic classification (0-4) provides a common language and conceptual decision-making support to modulate the use of EBD, subtractive surgeries and regenerative strategies more rationally, overcoming the “structural blindness’ of existing descriptive scales.

The phenotypic thresholds proposed here remain preliminary and should be considered a starting point for research, not a definitive standard. Prospective multicenter studies—correlating MAALS values with complication rates, validated PROMs (BODY-Q Arms) and independent long-term aesthetic assessments—are necessary to confirm, refine and potentially further stratify the system; such studies must specifically include the assessment of inter-operator reproducibility across multiple centers and correlation with three-dimensional imaging systems. Integration with AI-based segmentation algorithms and SWE represents the natural direction of development, with the ultimate aim of making MAALS a standardized, reproducible and widely adoptable diagnostic tool for the objective assessment of upper limb laxity, with a view to extending the compositional model to other body segments with high aesthetic demand.

## Data Availability

This article does not generate or analyse original datasets. The literature data cited are accessible via the referenced publications.
